# Validation and Evaluation of Reference Genes for Quantitative Real-Time PCR Analysis in *Mythimna loreyi* (Lepidoptera: Noctuidae)

**DOI:** 10.3390/insects15030185

**Published:** 2024-03-10

**Authors:** Liuyang Wang, Chaoxia Yang, Qingyu Liu, Xiaofang Zhang, Xiangdong Mei, Tao Zhang, Jun Ning

**Affiliations:** 1 Plant Protection Institute, HAAFS/Key Laboratory of IPM on Crops in Northern Region of North China, Ministry of Agriculture and Rural Affairs, China/IPM Innovation Center of Hebei Province/International Science and Technology Joint Research Center on IPM of Hebei Province, Baoding 071000, China; wliuyang1008@163.com (L.W.); zxfang224@163.com (X.Z.); 2 State Key Laboratory for Biology of Plant Diseases and Insect Pests, Institute of Plant Protection, Chinese Academy of Agricultural Sciences, Beijing 100193, China; ycx930501@163.com (C.Y.); lqy19950716@163.com (Q.L.); xdmei@ippcaas.cn (X.M.); 3 Innovation Center of Pesticide Research, Department of Applied Chemistry, College of Science, China Agricultural University, Beijing 100193, China

**Keywords:** *Mythimna loreyi*, qRT-PCR, gene stability, reference genes, normalization

## Abstract

**Simple Summary:**

The utilization of reference genes is very important for normalizing quantitative real-time PCR (qRT-PCR) expression data across various organisms subjected to varying experimental conditions. However, the stability and efficacy of reference genes are constrained to particular conditions. As a major migratory agricultural pest, *Mythimna loreyi* is widely distributed in Asia, Africa, Europe, and Australia. In this study, we conducted validation and evaluation of 13 reference genes, namely *RPL10*, *RPL27*, *RPL32*, *RPS3*, *TATA−box*, *GAPDH*, *AK*, *Actin*, *EF*, *α−tubulin*, *SOD*, *18S rRNA*, and *FTZ−F1*, to normalize qRT-PCR data for *M. loreyi*. Our results indicate that *RPL27* and *RPL10* are the best reference genes for the developmental stage, tissues, and adult age, *EF* and *RPS3* are the best for mating status, *AK* and *RPL10* are the best for temperature treatments, *RPL27* and *FTZ-F1* are the best for larva diet, and *EF* and *RPL27* are the best for adult diet treatments. Additionally, the validation of the reference genes was conducted through the utilization of a qRT-PCR-based gene expression analysis of two specific genes, namely *MlorPBP2* and *MlorGST1*. This study is essential for the accurate normalization of qRT-PCR data in *M. loreyi*, concurrently providing a valuable approach that can be applied to other insect species.

**Abstract:**

Quantitative real-time PCR (qRT-PCR) is a widely applied technique for accurately assessing the expression of target genes. In practice, the evaluation of gene expression requires appropriate reference genes. To screen reliable reference genes for evaluating gene expression via qRT-PCR in *Mythimna loreyi*, a notorious migratory pest across Asia, Africa, Europe, and Australia, we assessed the expression stability of 13 candidate reference genes in *M. loreyi* using the ΔCt method, BestKeeper, Normfinder, GeNorm, and the web-based comprehensive platform RefFinder. These reference genes include *RPL10*, *RPL27*, *RPL32*, *RPS3*, *TATA−box*, *GAPDH*, *AK*, *Actin*, *EF*, *α−tubulin*, *SOD*, *18S rRNA*, and *FTZ−F1*, which is frequently employed in Lepidoptera insects. Our findings revealed that the performance of the candidate reference gene depended on experimental conditions. Specifically, *RPL27* and *RPL10* were the most suitable for evaluating expression changes across developmental stages, tissues, and adult ages. The optimal reference genes were recommended in specific experiment conditions, for instance, *EF* and *RPS3* were recommended for mating status, *AK* and *RPL10* were recommended for temperature treatments, *RPL27* and *FTZ*−*F1* were recommended for larva diet, and *EF* and *RPL27* were recommended for adult diet treatments. Additionally, expression profiles of pheromone-binding protein 2 (*MlorPBP2*) and glutathione S-transferase (*MlorGST1*) were used to validate the reference genes. This study provides reference genes for the accurate normalization of qRT-PCR data, laying the groundwork for studying the expression of target genes in *M. loreyi*.

## 1. Introduction

Quantitative real-time PCR (qRT-PCR) is a pivotal molecular technique. Due to its high sensitivity, reliability, and specificity, qRT-PCR is widely applied in quantitatively analyzing functional gene expression [1]. To ensure precision and reliability in outcomes, an appropriate reference gene should be carefully evaluated and selected for normalizing gene expression and mitigating errors stemming from experimental variations, including sample quantities, RNA quality and quantity, and PCR efficiency [2,3,4,5,6].

The reference genes most commonly utilized are classified as housekeeping genes, for example ribosomal RNA (*18S rRNA*), glyceraldehyde-3-phosphate dehydrogenase (*GAPDH*), TATA−box-binding protein (*TATA−box*), superoxide dismutase (*SOD*), alpha−tubulin (*α−tubulin*), elongation factor 1α (*EF*), *Actin*, arginine kinase (*AK*), and ribosomal protein [4]. Numerous studies indicate that the levels of expression of these housekeeping genes can vary significantly under various experimental conditions [6,7]. In non-model species, reference gene identification often relies on the orthologous sequence of well-established housekeeping genes from model insect species due to limited genetic and genome information. Misuse of conventional housekeeping genes as reference genes in non-model insects may introduce deviations or errors [5,8,9,10]. Hence, it is crucial to carefully choose appropriate reference genes according to the specific experimental conditions [3].

The loreyi leafworm, *Mythimna loreyi* Walker (Lepidoptera: Noctuidae), an invasive noctuid indigenous to East Asia, is prevalent in Africa, Australia, and numerous Asian countries [11]. Its larvae feed on diverse plant species, including rice, wheat, maize, sugarcane, barley, and sorghum [12,13]. Commonly, *M. loreyi* coexists with *M. separata* in China, increasing its threat to crop production [14]. To reduce the yield losses resulting from their damage, many studies have focused on the migratory patterns and regulatory mechanisms of *M. loreyi* and *M. separata* [15,16,17]. In recent years, the emergence of next-generation sequencing techniques has facilitated a multitude of investigations aimed at discerning and choosing genes associated with olfaction, sex pheromone biosynthesis, and migratory behavior in this specific species [18,19,20,21]. However, the optimal reference genes in *M. loreyi* under specific conditions are inconclusive. It is almost impossible that a singular “universal” reference gene could be applicable to all experimental conditions, even within one species [5,6,10,22,23,24]. Consequently, the selection and evaluation of reference genes in *M. loreyi* hold significant significance.

In this study, we validated and evaluated the suitability of 13 commonly utilized reference genes for normalizing qRT-PCR data for *M. loreyi*, including ribosomal protein L10 (*RPL10*), ribosomal protein L27 (*RPL27*), ribosomal protein L32 (*RPL32*), ribosomal protein S3 (*RPS3*), *TATA−box*, *GAPDH*, *AK*, *Actin*, *EF*, *α−tubulin*, *SOD*, *18S rRNA*, and Fushi tarazu transcription factor 1 (*FTZ−F1*). Further, we accessed the most appropriate reference genes for qRT-PCR analysis in *M. loreyi* under various conditions through the utilization of five statistical techniques (the ΔCt method, BestKeeper, Normfinder, and GeNorm) for standardization. Finally, we selected two target genes, namely pheromone-binding protein 2 (*MlorPBP2*) and glutathione S-transferase (*MlorGST1*, GenBank: KAJ8724239.1), which play crucial roles in detoxification and olfaction [25,26], for validation. Our results potentially contribute to improving the normalization of *M. loreyi* qRT-PCR data.

## 2. Materials and Methods

### 2.1. Insect Rearing

Approximately 200 larvae of *M. loreyi* were collected in September 2021 from cornfields of the Agricultural Experiment Station of the Institute of Plant Protection, Hebei Academy of Agriculture and Forestry Sciences (38.95° N, 115.45° E), Baoding, China. The larvae of *M. loreyi* were reared on corn plants at 26 ± 2 °C in rearing boxes (300 cm × 120 cm × 80 cm) with a relative humidity of 60 ± 10% and a light/dark cycle of 14:10 h. The pupae were separated by sex and kept in individual plastic containers (diameter 2 cm, 10 cm). The moths were given a 10% sucrose solution after they emerged. Sample collection began with the sixth-generation insect.

### 2.2. Sample Collection under Various Biotic Factors

Developmental stage: The samples utilized in this study included 200 eggs, 30 first instars, 10 second instars, 5 third instars, and 2 individuals of the remaining stages (fourth–sixth instars, pupae, and 3-day-old female and male adults). The samples were collected in 1.5 mL microcentrifuge tubes and promptly frozen in liquid nitrogen before being stored at −80 °C. Each treatment was replicated three times.

Tissue: 3-day-old adults were dissected for heads (10 individuals), thoraxes (2 individuals), abdomens (2 individuals), legs (30 individuals), wings (20 individuals), female antennas (50 pairs), and male antennas (50 pairs) in a pre-cooled phosphate-buffered saline (PBS) solution. 21 samples were collected, each consisting of three biological replicates from seven tissues.

Adult age: Female moths at different ages (1 day old, 3 day old, 5 day old, 7 day old, and 9 day old) with three replicates were selected and promptly stored at −80 °C.

Mating status: Three mated and unmated male adults and female adults were collected separately to examine the impact of mating status.

### 2.3. Sample Collection under Various Abiotic Conditions

Temperature: Fifteen three-day-old female adults were exposed to five different temperatures from 10 °C to 30 °C (10 °C, 15 °C, 20 °C, 25 °C, and 30 °C) for two hours, with three biological replicates. Subsequently, they were promptly stored at −80 °C.

Larva diet: *M. loreyi* larvae were raised on corn seedlings or artificial feed [27]. Subsequently, five third-instar, one five-instar, one male adult, and one female adult were collected from each experimental condition, with three replicates.

Adult diet: In insects, supplemental sugar increases the trehalose concentration in pheromone glands, promoting sex pheromone biosynthesis and facilitating successful mating [28]. Newly emerged female moths were gathered and fed 10% monosaccharides (glucose, fructose, and galactose), 10% disaccharides (maltose, sucrose, and lactose), or water. Subsequently, 30 pheromone glands were collected separately at the 72 h mark, with three replicates for each treatment.

### 2.4. RNA Extraction and cDNA Synthesis

Total RNA was isolated from each sample using TRIzol reagent (TransGen Biotech, Beijing, China) following the manufacturer’s instructions. RNA concentration and purity were assessed using NanoDrop One Spectrophotometer (Thermo Fisher, Fremont, CA, USA). All samples with an OD_260nm_/OD_280nm_ value of 1.9–2.2 were used for subsequent experiments. In addition, RNA integrity was evaluated through 1.2% agarose gel electrophoresis. First-strand cDNAs were synthesized by reverse-transcribing 1 μg of total RNA using All-in-One Super Mix for qPCR Reagent Kit, manufactured by TransGen Biotech in Beijing, China.

### 2.5. Candidate Reference Gene Selection and Primer Design

The sequences of 13 candidate reference genes commonly used in studies of Lepidoptera insects, including *RPL10*, *RPL27*, *RPL32*, *RPS3*, *TATA−box*, *GAPDH*, *AK*, *Actin*, *EF*, *α−tubulin*, *SOD*, *18S rRNA*, and *FTZ−F1*, were obtained from the genome data of *M. loreyi* (GenBank accessions: GCA_029852875.1). The primer pairs utilized for amplification were meticulously designed using the software Primer Premier 6.0 following the principles for designing qRT-PCR primers. The selected primer pairs were synthesized by Sangon Biotechnology Co., Ltd. (Shanghai, China). The purified PCR products were used as the initial templates to create standard curves for determining primer amplification efficiency. All templates were subjected to two-fold serial dilution, resulting in a total of five gradients. Table 1 shows the sequences and lengths of primer pairs for the 13 reference genes and their amplification efficiency.

### 2.6. qRT-PCR Analysis

qRT-PCR was carried out in a 20 μL system, comprising 10 μL 2 × TransStart Tip Green qPCR SuperMix (TransGen Biotech, Beijing, China), 0.5 μL of each gene-specific primer (Table 1), 1 μL of cDNA template, and 8 μL of ddH_2_O, on QuantStudio 3 System (Thermo Fisher Scientific Inc., Waltham, MA, USA). All tests had three biological replicates and three technical replicates. The PCR conditions consisted of 5 s of initial denaturation at 94 °C, followed by 40 cycles of 15s denaturation at 94 °C and 10 s extension at 72 °C. Subsequently, a melting curve analysis was performed to assess the specificity of the primers.

### 2.7. Statistical Analysis

The stability of the 13 candidate reference genes was evaluated using the ΔCt method [29], BestKeeper [30], NormFinder [31], and GeNorm [32]. Subsequently, the most suitable reference genes were identified using RefFinder (https://blooge.cn/RefFinder/, accessed on 26 November 2023) [33]. The optimal number of reference genes for normalization was determined using GeNorm, taking into account the V value. A ratio of V_n/n+1_ exceeding 0.15 indicated the need for an additional reference gene, thereby determining the optimal number of reference genes as n + 1.

### 2.8. Validation of Reference Genes

Two genes, *MlorPBP2* and *MlorGST1*, were selected based on available literature and genome data of *M. loreyi* to validate the two most stable reference genes (*RPL27* and *RPL10*) and the most varying reference gene (*Actin*) identified [18,20,21]. The primer sequences utilized in this study were as follows: *MlorPBP2*, F: 5′-ATGGTGCTCCATCGATCG-3′, R: 5′-TTAAATTTCAGCCAAGACTTCTC-3′; *MlorGST1*, F: 5′-CAACTGCTCAACACATTCC-3′, R: 5′-AGGTCTTCACTGTCTCGTA-3′. qRT-PCR data were analyzed using the 2^−ΔΔCt^ method [34], with three replicates for each experiment. Statistical analysis was performed employing one-way analysis of variance (ANOVA) followed by Tukey’s test using SPSS Statistics version 27.

## 3. Results

### 3.1. Primer Specificity and Efficiency

The amplification of all 13 candidate reference genes resulted in a single band of the anticipated size on 1.2% agarose gel and a single peak in the melting curve analysis, confirming the specificity of the primer pairs (Figures S1 and S2). Additionally, all amplifications achieved reasonable efficiencies between 95.3 and 108.6% with a high correlation coefficient (R^2^ ≥ 0.990) (Table 1 and Figure S3).

### 3.2. Expression Profiles of Candidate Reference Genes

**Figure 1:**
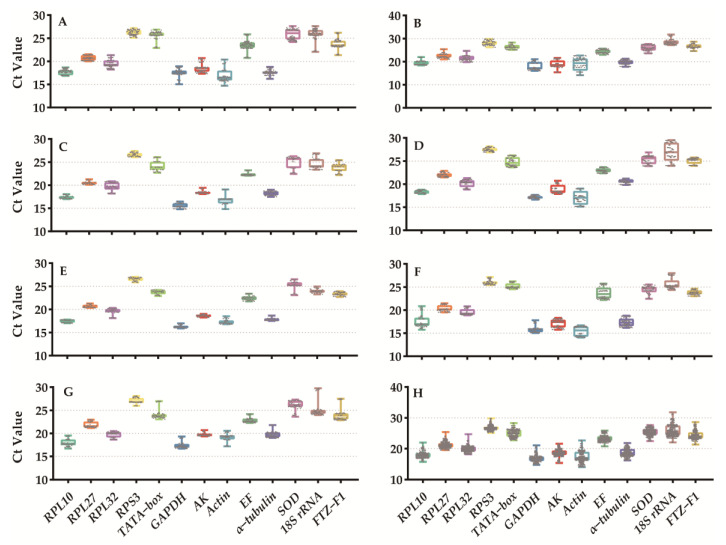
Expression profiles of 13 candidate reference genes of *Mythimna loreyi* in different treatments. (**A**) Developmental stage, (**B**) tissue, (**C**) adult age, (**D**) mating status, (**E**) temperature, (**F**) larva diet, (**G**) adult diet, and (**H**) total. The box plots depict the median (horizontal line), 25 and 75% quartiles (box), and min and max values (whiskers).

Among the 13 reference genes, *GAPDH* exhibited the lowest Ct value, indicating its high expression as a reference gene. *Actin*, *RPL10*, *AK*, *α−tubulin*, *RPL32*, *RPL27*, *EF*, *FTZ−F1*, *TATA−box*, *SOD*, *18S rRNA*, and *RPS3* were ranked in terms of their abundance. Notably, *18S rRNA*, *Actin*, and *FTZ−F1* displayed the highest variation in expression, followed by *RPL32* > *GAPDH* > *RPL10* > *AK* > *RPL27* > *α−tubulin* > *TATA−box* > *SOD* > *EF* > *RPS3* (Figure 1). Additionally, our results indicated that the expression of certain reference genes varied depending on the experimental treatments. For example, *FTZ−F1* showed lower variation (approximately one cycle) for adult age but higher variation (more than four cycles) for developmental stage, tissue, and diet treatment (Figure 1H).

### 3.3. Stability of Reference Genes across Biotic Factors

**Figure 2:**
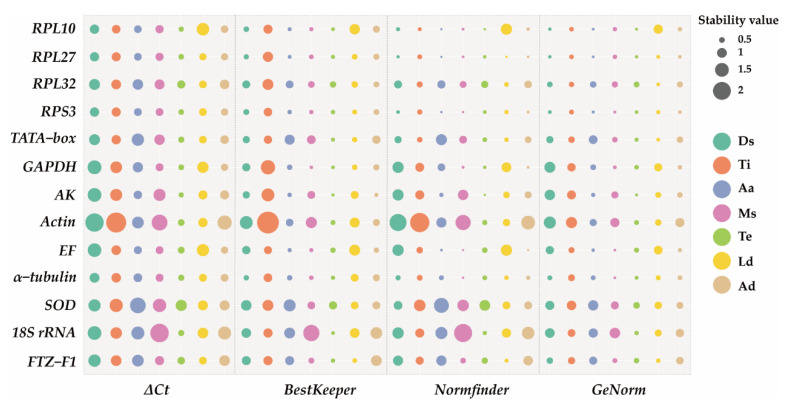
The stability of the 13 candidate reference genes evaluated using four different methods: the ΔCt method, BestKeeper, NormFinder, and GeNorm. The bubble size represents the geomean value; the larger the bubble, the larger the value (lower value indicates higher stability). Ds: developmental stage; Ti: tissue; Aa: adult age; Ms: mating status; Te: temperature; Ld: larva diet; Ad: adult diet.

**Figure 3:**
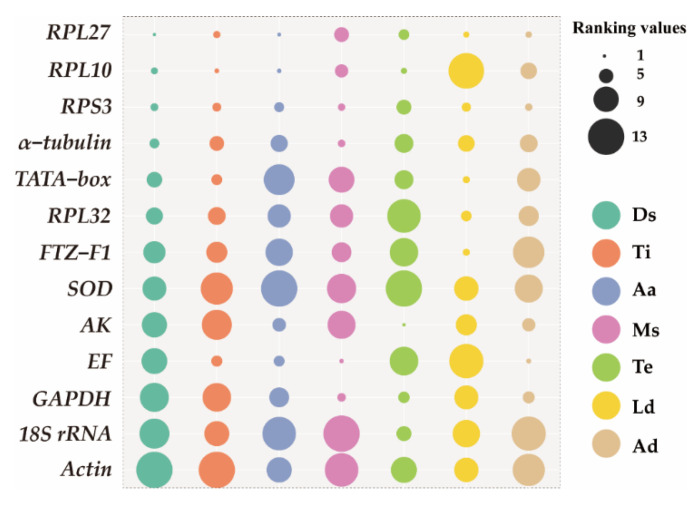
Expression stability of 13 candidate reference genes of *Mythimna loreyi* in different treatments determined using RefFinder. The bubble size represents the geomean value. A lower geomean value indicates a more stable expression. Ds: developmental stage; Ti: tissue; Aa: adult age; Ms: mating status; Te: temperature; Ld: larva diet; Ad: adult diet.

**Figure 4:**
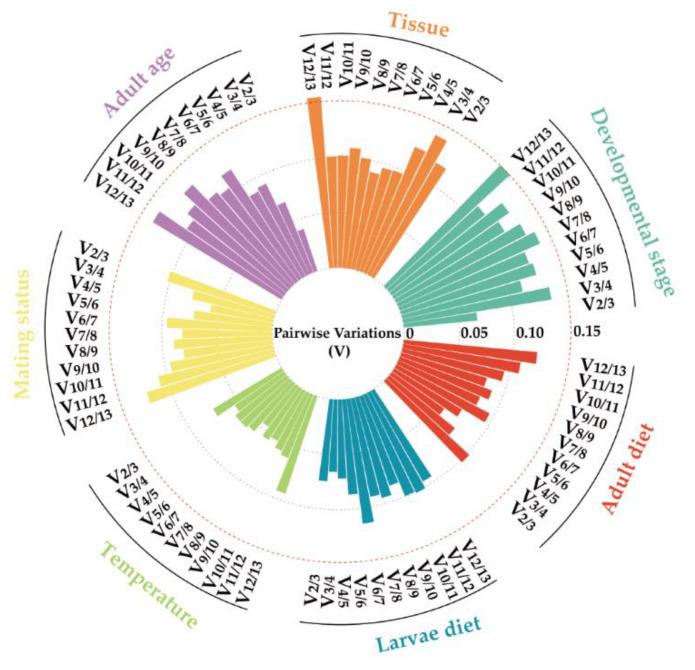
The determination of the ideal quantity of reference genes for the normalization of *Mythimna loreyi* under different treatment conditions. GeNorm v3.4 software was used to calculate the pairwise variation (V) value, which was used as a metric to determine the optimal number of reference genes in the qRT-PCR analysis. A pairwise variation value below 0.15 was considered an indication of a sufficient number (n) of reference genes for effectively normalizing the expression of the target gene. The red dashed line on the graph represents the threshold of 0.15 for the pairwise variation.

The analysis of qRT-PCR results of samples at different developmental stages using the ΔCt method and NormFinder revealed that *RPL27*, *RPS3*, and *RPL10* exhibited the highest stability as reference genes. BestKeeper and GeNorm results revealed that *RPL27*, *RPS3*, and *α−tubulin* were the most suitable reference genes (Figure 2). Consistently, four methods indicated that *Actin* was the least stable reference gene (Figure 2). RefFinder analysis ranked the expression stability as follows: *RPL27* > *RPL10* > *RPS3* > *α−tubulin* > *TATA−box* > *RPL32* > *FTZ−F1* > *SOD* > *AK* > *EF* > *GAPDH* > *18S rRNA* > *Actin* (Figure 3). GeNorm analysis revealed a pairwise variation, V_2/3_, value (0.067) below the threshold of 0.15 (Figure 4), suggesting that utilizing *RPL27* and *RPL10* as reference genes at different developmental stages was the optimal normalization strategy (Figure 3).

qRT-PCR results of samples from various tissues using the ΔCt method, GeNorm, and NormFinder showed that *RPL10* was the most stable reference gene (Figure 2). RefFinder analysis showed a stable order: *RPL10* > *RPL27* > *RPS3* > *TATA−box* > *EF* > *α−tubulin* > *RPL32* > *FTZ−F1* > *18S rRNA* > *GAPDH* > *AK* > *SOD* > *Actin* (Figure 3). GeNorm analysis yielded a pairwise value, V_2/3_, below 0.15 (0.119) (Figure 4), suggesting that *RPL10* and *RPL27* were sufficient for reliable normalization across diverse tissues (Figure 3).

For adult age, *RPL27* and *RPL10* emerged as the most stable genes according to the ΔCt method, GeNorm, BestKeeper, and NormFinder. Conversely, *SOD* and *18S rRNA* were deemed the most unstable genes across all algorithms (Figure 2). RefFinder analysis demonstrated *RPL27* as the most stable reference gene, followed by *RPL10*, *RPS3*, *EF*, *AK*, *α−tubulin*, *GAPDH*, *RPL32*, *Actin*, *FTZ−F1*, *TATA−box*, *18S rRNA*, and *SOD* (Figure 3). GeNorm analysis revealed a pairwise value, V_2/3_, below 0.15 (Figure 4). All these analyses indicated that *RPL27* and *RPL10* were the most suitable reference genes for normalizing qRT-PCR data across various adult age groups (Figure 3).

Based on the NormFinder and GeNorm analyses conducted on different mating statuses, *EF* and *SOD* were regarded as the most stable genes. However, when employing the ΔCt method, *RPS3* and *EF* emerged as the two most stable genes. On the other hand, analysis using BestKeeper identified *GAPDH* and *EF* as the most stably expressed genes (Figure 2). RefFinder analysis indicated *EF* as the most stable gene under the influence of mating status, followed by *RPS3*, *α−tubulin*, *GAPDH*, *RPL10*, *RPL27*, *FTZ−F1*, *RPL32*, *TATA−box*, *AK*, *SOD*, *Actin*, and *18S rRNA* (Figure 3). GeNorm analysis showed a pairwise variation value, V_2/3_, below 0.15 (Figure 4), suggesting that the combination of *EF* and *RPS3* was suitable for effectively normalizing qRT-PCR data under varying mating statuses (Figure 3).

### 3.4. Stability of Reference Genes under Abiotic Stresses

*AK* and *RPL10* were identified as the most stable genes across different temperature treatments, according to the ΔCt method, NormFinder, and GeNorm analyses. BestKeeper found *AK* and *GAPDH* to be the most suitable reference genes. (Figure 2). RefFinder analysis further ranked the stability as follows: *AK* > *RPL10* > *RPL27* > *GAPDH* > *RPS3* > *18S rRNA* > *α−tubulin* > *TATA−box* > *Actin* > *FTZ−F1* > *EF* > *RPL32* > *SOD* (Figure 3). GeNorm analysis indicated a pairwise value, V_2/3_, below 0.15 (Figure 4), implying that utilizing *AK* and *RPL10* was adequate for dependable normalization under different temperature treatments (Figure 3).

*RPL27* was identified as the most stable gene for larva diet using the ΔCt method and NormFinder analysis, while *FTZ-F1* and *RPL32* were recognized as stable reference genes under BestKeeper and GeNorm, respectively (Figure 2). RefFinder analysis showed that the stability reference gene ranking under different larva diet treatments is as follows: *RPL27* > *FTZ−F1* > *TATA−box* > *RPS3* > *RPL32* > *α−tubulin* > *AK* > *GAPDH* > *Actin* > *SOD* > *18S rRNA* > *EF* > *RPL10* (Figure 3). GeNorm analysis showed a V_2/3_ value below 0.15 (Figure 4), suggesting the necessity of utilizing two reference genes for normalization, with *RPL27* and *FTZ−F1* being the most suitable reference gene combinations for different larva diet treatments (Figure 3).

For adult diet, *RPL27* was identified as the most stable reference gene according to the GeNorm and NormFinder analyses, while *EF* and *AK* were found to be the most stable genes using the ΔCt method and BestKeeper (Figure 2). All four methods revealed that *18S rRNA* and *Actin* were the least stable genes (Figure 2). RefFinder analysis ranked the reference genes’ stability as follows: *EF*, *RPL27*, *RPS3*, *GAPDH*, *AK*, *RPL10*, *α−tubulin*, *RPL32*, *TATA−box*, *SOD*, *FTZ−F1*, *Actin*, and *18S rRNA* (Figure 3). GeNorm analysis showed a pairwise variation value, V_2/3_ (0.102), below 0.15 (Figure 4), indicating that both *EF* and *RPL27* could serve as suitable normalization factors under different adult diet treatments (Figure 3).

### 3.5. Validation of Candidate Reference Genes

**Figure 5:**
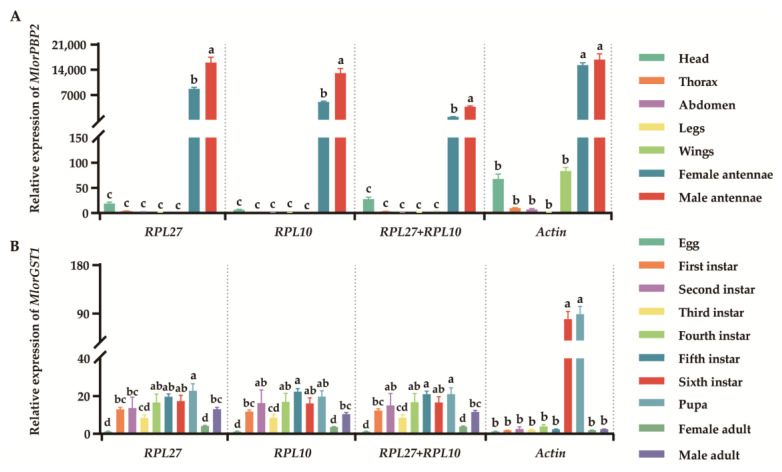
Validation of the gene stability measures. (**A**) *MlorPBP2* expression levels in various tissues. The relative expression level of *MlorPBP2* was standardized using the most appropriate reference genes (*RPL27* and *RPL10*), as well as the least suitable reference gene (*Actin*). (**B**) *MlorGST1* expression levels in different adult diet treatments. The relative expression level of *MlorGST1* was standardized using the most appropriate reference genes (*RPL27* and *RPL10*), as well as the least suitable reference gene (*Actin*). The mean ± SE values, derived from three distinct biological replicates, were reported as the outcomes. These outcomes were assessed utilizing a one-way analysis of variance (ANOVA) and subsequently subjected to Tukey’s multiple comparison test. Different letters show significant differences (*p* < 0.01).

To ascertain the appropriateness of the suggested reference genes, we assessed the mRNA expression of the target genes *MlorPBP2* and *MlorGST1* in diverse tissues and samples under different developmental stages using the worst and best candidate genes or gene combinations for normalization. Inconsistent expression patterns of *MlorPBP2* and *MlorGST1* in various tissues and under different developmental stages were observed when they were normalized with the most or least stable reference genes (Figure 5). For instance, *MlorPBP2* was expressed the most in male antennae when employing *RPL27* and *RPL10*, the most stable reference genes. However, when using the less stable gene *Actin* (F_1,2_ = 1.099, *p* = 0.188) as the reference gene, the expression level of *MlorPBP2* in male antennae was not significantly different from that in females (Figure 5A). Similarly, significant differences were observed in *MlorGST1* expression in developmental stage samples under the fifth instar and sixth instar when using *Actin* (F_1,2_ = 11.037, *p* < 0.001) for normalization, but not when using others (Figure 5B), emphasizing the impact of reference gene combinations on target gene normalization.

## 4. Discussion

qRT-PCR is a crucial methodology for examining relative mRNA levels of target genes across diverse biological systems [35]. However, its accuracy predominantly hinges upon the internal control, commonly known as a reference or “housekeeping” gene [3]. Massive investigations have substantiated the significant variability in the expression of reference genes across distinct cell types, tissues, and experimental circumstances [5,6,13,23,36]. Hence, it is important to confirm the stability of reference genes across different experimental conditions before using them for gene expression normalization.

Previous qRT-PCR studies on *M. loreyi* were conducted with a preference for common insect reference genes [18,19,20,21] such as *Actin*, *EF*, *GAPDH*, and *RPS3*; however, our research found that neither *Actin* nor *GAPDH* was suitable as an optimal reference under diverse abiotic and biotic factors, with *Actin* exhibiting particularly unfavorable performance (Figure 3). *RPS3* and *EF* were suitable as reference genes solely in terms of distinct mating status and adult diet treatments, displaying inadequate stability in alternative circumstances (Figure 3). Likewise, for all samples, *RPS3* was regarded as the most stable gene according to RefFinder analysis (Figure S4). It should be noted that the expression levels of conventional reference genes in various insect species exhibit significant variation, and no single gene maintained consistent expression levels across all conditions [37,38]. Therefore, this study utilized four different software programs to evaluate the expression stability of the 13 potential reference genes across seven distinct experimental conditions.

Our results indicate that the ΔCt method, BestKeeper, NormFinder, and GeNorm yielded distinct stability rankings for the 13 reference genes. Specifically, the ΔCt method and NormFinder identified *EF* as the most stable reference gene across various adult diet treatments, while BestKeeper and GeNorm recommended *AK* and *RPL27* as the most stable reference genes, respectively (Figure 2). These divergent findings could potentially be ascribed to the distinct algorithms [4,39]. To reconcile these differences, we employed the online tool RefFinder to generate an ultimate stability ranking. The results showed *RPL27* as the most stably expressed gene across various developmental stages, adult ages, and larval diet treatments, with *RPL10* being the most stable gene across different tissues, *EF* as the optimal reference gene across different mating statuses and adult diets, and *AK* exhibiting the highest stability under different temperatures (Figure 3).

Ribosomal proteins (RPs), as fundamental constituents of ribosomes, are considered the most extensively conserved proteins across all biological samples [23,40]. Therefore, their coding genes have been extensively employed as reference genes in insect molecular research on gene expression regulation over the preceding decade [3]. However, our study revealed variations in its expression levels across different treatments. For instance, *RPL32* exhibited stability under larva diets but exhibited the poorest performance under diverse temperature conditions. *RPL10* was stable under various conditions except for larval diets (Figure 3). Consistent with our findings, other studies have shown that *RPS11* expression is stable across various developmental stages in *Tuta absoluta* but is the lowest in diverse adult tissues [41]. In *Cnaphalocrocis medinalis*, *RPL13* emerges as the most stable ribosomal protein across different adult ages but becomes the most unstable one in larval tissues and in larvae under temperature treatments [42]. These investigations suggest that the expression of ribosomal proteins varies significantly under different experimental conditions.

rRNA genes, encoding crucial constituents of ribosomes, have been widely used as reference genes in studies on insects, such as *Sesamia inferens* [43], *Cotesia chilonis* [44], and *Athetis dissimilis* [45]. However, our results revealed that the expression of *18S rRNA* was unstable under both biotic and abiotic stresses, particularly under diverse developmental and mating states and under different adult diet treatments (Figure 3). Our findings align with the previous observations [6].

*Actin*, a gene responsible for encoding a prominent structural protein across numerous cell types, is widely used as the optimal reference gene for qRT-PCR analysis [3]. However, our data revealed *Actin* as an unsuitable reference gene in various developmental stages of *M. loreyi*, consistent with the findings for other insect species, such as *C. hemipterus* [5], *Phenacoccus solenopsis* [46], *H. armigera* [22], *Aquatica leii* [9], and *S. litura* [47]. However, *Actin* expression remains stable in several other insects, including *M. separata* [23], *Chortoicetes terminifera* [48], and *Apis mellifera* [49].

*EF*, a recognized housekeeping gene, is ranked as the most commonly used gene on the ICG website (https://ngdc.cncb.ac.cn/icg/, accessed on 2 December 2023) and has been utilized as a reference gene for studies on various species and under different experimental conditions [5,8,23,24,50]. Our results indicated that it was one of the two most stable genes under different mating statuses and adult diet treatments but that it was not stable under other experimental conditions (Figure 3). The varying performances of *MlorEF* observed in this study underscore the absence of a universal reference gene that can be universally applied to all species or across diverse experimental conditions.

It is widely acknowledged that employing a combination of multiple reference genes to normalize gene expression in qRT-PCR analysis could yield more precise and reliable expression patterns compared with those obtained when utilizing a solitary gene [3,4,51]. GeNorm analysis not only assesses the stability of reference genes but also computes the most suitable combination of reference genes for a specific condition [32]. Following GeNorm guidelines, a pairwise variance value, V_2/3_, below 0.15 indicates that the optimal number of combinations is 2. In our study, the pairwise variance value (V_2/3_) across different conditions fell below 0.15 (Figure 4), necessitating the use of two reference genes for accurate gene expression analysis.

Massive studies have indicated that the selection of reference genes can significantly impact biological findings [3,40,52]. The utilization of an unstable reference gene may lead to an inaccurate representation of the target gene’s expression pattern, resulting in misinterpretations [4,5,23,46]. In our investigation, the expression patterns of *MlorPBP2* and *MlorGST1* varied across different tissues and developmental stages when normalized with both stable and unsuitable reference genes (Figure 5). These findings underscore the potential for misleading outcomes in relation to the functionality of a target gene when a reference gene is arbitrarily chosen.

## 5. Conclusions

Our study systematically investigated and validated 13 potential reference genes for normalizing qRT-PCR data in *M. loreyi* under various abiotic and biotic conditions. To our knowledge, this research represented the first validation of reference genes in *M. loreyi* for qRT-PCR data normalization. Our findings showed that the most suitable reference gene combinations were as follows: *RPL27* and *RPL10* for developmental stages, tissues, and adult ages, *EF* and *RPS3* for mating statuses, *AK* and *RPL10* for temperature treatments, *RPL27* and *FTZ−F1* for larva diets, and *EF* and *RPL27* for adult diet treatments. The evaluation of stable reference genes could provide a foundational framework for the accurate and robust utilization of qRT-PCR in *M. loreyi*, enabling a precise and comprehensive analysis of gene expression.

## Figures and Tables

**Table 1:** Reference genes and specific primers used for qRT-PCR analysis of *Mythimna loreyi*.

Gene	Accession No.	Primer Sequences (5′-3′, F/R)	Amplicon Length (bp)	Tm (°C)	Efficiency (%)	R^2^
*RPL10*	KAJ8725396.1	ACCTGGTGTCTGATGAGTA	295	55	97.2	0.994
		ATGACCTGAGCCTTCCAA				
*RPL27*	KAJ8722287.1	TGAAGAACTACGACGAAGG	187	55	97.7	0.990
		TCAACTGAGTAGCGAGTG				
*RPL32*	KAJ8705474.1	CAATCGGATCGCTATGACA	339	55	108.6	0.995
		TTATTCGTTCTCCTGGCTAC				
*RPS3*	KAJ8720489.1	GTCCGCAAGAGGTTCAAT	339	55	102.3	0.997
		CTTAATTCCGAGCACTCCT				
*TATA−box*	KAJ8730825.1	TCATACTCCTGTCCACTGT	180	55	107.0	0.992
		TCTGTTCCACTCACCATTG				
*GAPDH*	KAJ8714943.1	GGCACAGTCGGTATAGAAG	283	55	97.9	0.997
		AGGAAGCGTTGGAGATGA				
*AK*	KAJ8736045.1	CTGGTGTCGGAATCTACG	108	55	102.3	0.995
		GCTTGTCGGTCTTCTTGA				
*Actin*	KAJ8708612.1	AATCGTGCGTGACATCAA	473	55	95.3	0.999
		ACTCGTCGTATTCCTCCTT				
*EF*	KAJ8731840.1	ACACAGCTCGGATACAGT	100	55	106.8	0.992
		GCATCAACCCAGTCGTTA				
*α−tubulin*	KAJ8707351.1	CGCTACCATCAAGACCAA	257	55	107.7	0.996
		ACTCTCCTTCCTCCATACC				
*SOD*	KAJ8723090.1	CGAGTAATTGCGGTGTCA	146	55	100.2	0.998
		CGTAGTCTTGCTCAGGTC				
*18S rRNA*	KAJ8712979.1	ATCCAAGCACAGATGACAG	464	55	103.5	0.998
		ACAACTACACCTCCATAGAAG				
*FTZ−F1*	KAJ8734564.1	TAACAGACGGCACATCAC	220	55	100.1	0.999
		TGTAAGGCACCAATGAGTT				

## Data Availability

The data presented in this study are available on request from the corresponding author.

## Supplementary Materials

The following supporting information can be downloaded at https://www.mdpi.com/article/10.3390/insects15030185/s1: Figure S1: The presence of a single amplicon of the desired size for each candidate reference gene observed through visualization on 1.2% agarose gel; Figure S2: Melting curve analysis of thirteen candidate reference genes; Figure S3: Standard curves of the thirteen candidate reference genes. Figure S4: Expression stability of 13 candidate reference genes of *Mythimna loreyi* in all samples determined using RefFinder.
