# Supplementary material for: Validation and Evaluation of Reference Genes for Quantitative Real-Time PCR Analysis in Mythimna loreyi (Lepidoptera: Noctuidae)
Source: Insects. 2024 Mar 10;15(3):185. doi: 10.3390/insects15030185 (PMC10970824; doi:10.3390/insects15030185)
Supplement: Supplementary file 1 [file insects-15-00185-s001.zip › insects-2867893-supplementary.pdf]

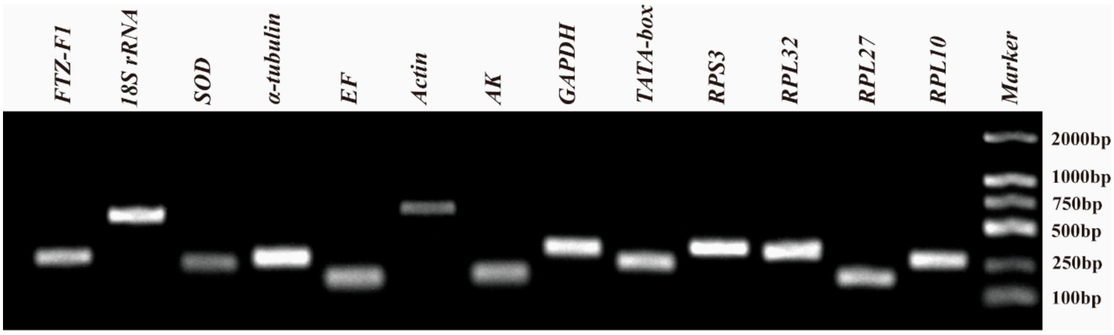

Figure S1. The presence of a single amplicon of the desired size for each candidate reference gene observed through visualization on 1.2% agarose gel.

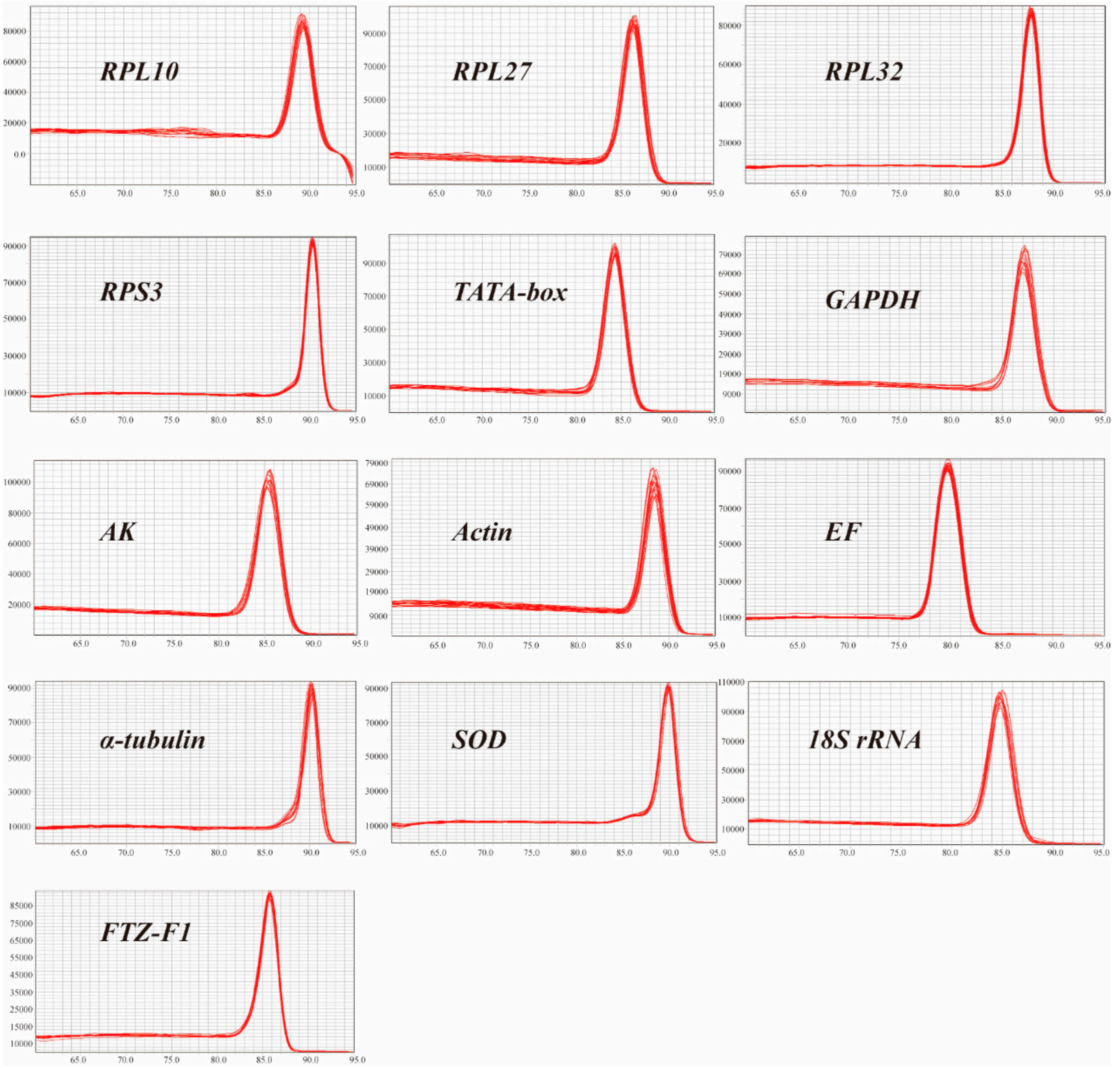

Figure S2. Melting curve analysis of thirteen candidate reference genes.

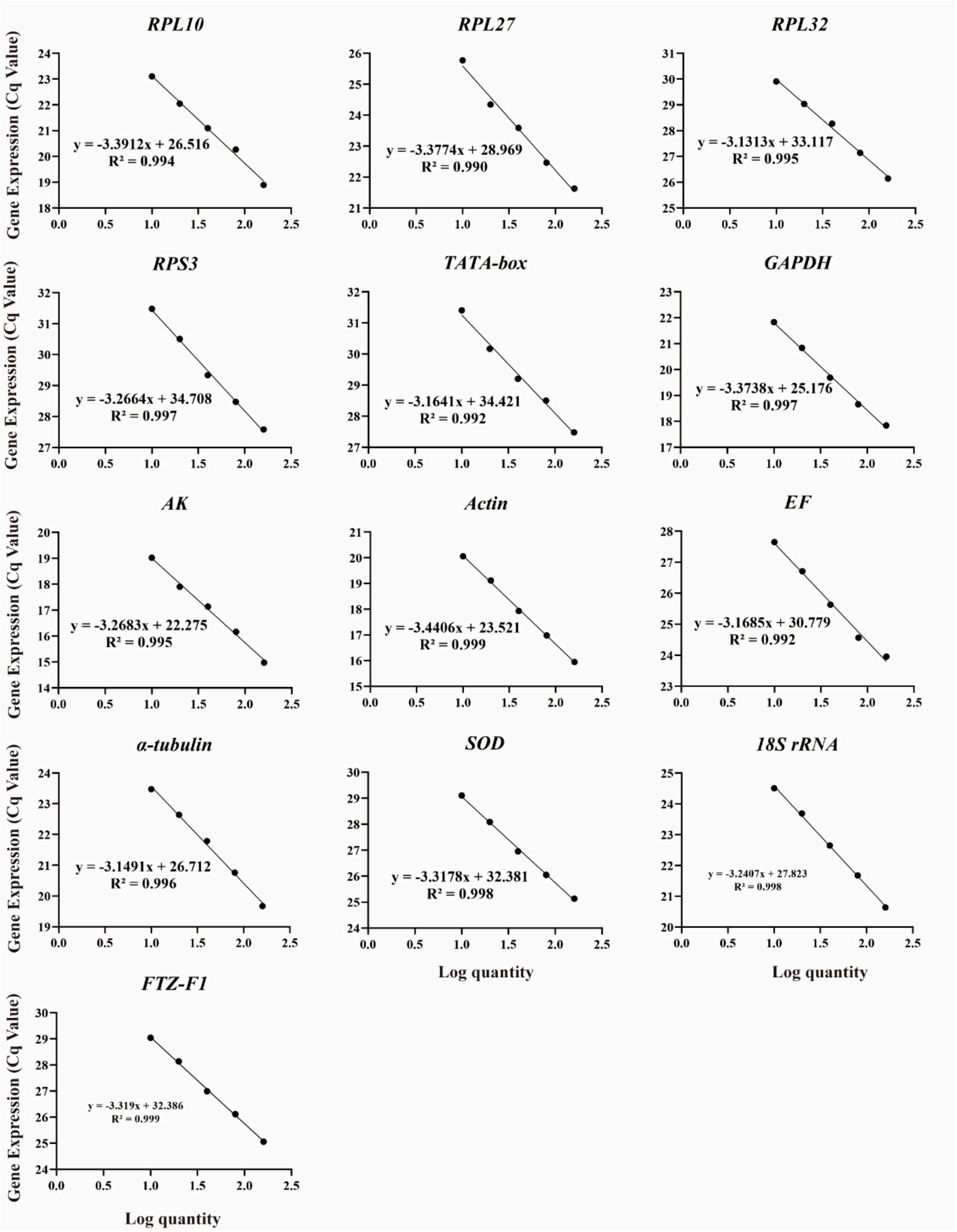

Figure S3. Standard curves of the thirteen candidate reference genes.

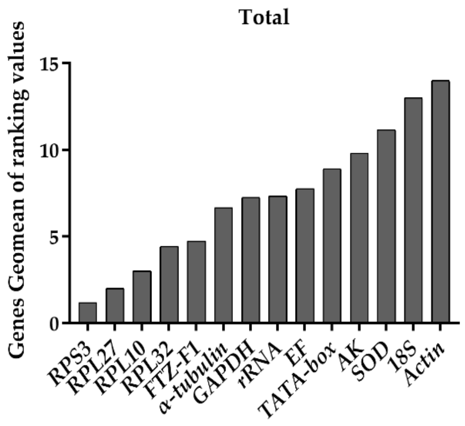

Figure S4. Expression stability of 13 candidate reference genes of *Mythimna loreyi* in all samples determined using RefFinder.
